# Anaerobic phloroglucinol degradation by *Clostridium scatologenes*

**DOI:** 10.1128/mbio.01099-23

**Published:** 2023-06-21

**Authors:** Yan Zhou, Yifeng Wei, Li Jiang, Xinan Jiao, Yan Zhang

**Affiliations:** 1 Jiangsu Key Laboratory of Zoonosis, Yangzhou University, Yangzhou, China; 2 Key Laboratory of Prevention and Control of Biological Hazard Factors (Animal Origin) for Agrifood Safety and Quality, Ministry of Agriculture of China, Yangzhou University, Yangzhou, China; 3 Jiangsu Co-Innovation Center for Prevention and Control of Important Animal Infectious Diseases and Zoonoses, Yangzhou University, Yangzhou, Jiangsu Province, China; 4 Singapore Institute of Food and Biotechnology Innovation, Agency for Science, Technology and Research (A*STAR), Singapore, Singapore; 5 Tianjin Key Laboratory for Modern Drug Delivery & High-Efficiency, Collaborative Innovation Center of Chemical Science and Engineering, School of Pharmaceutical Science and Technology, Tianjin University, Tianjin, China; 6 Frontiers Science Center for Synthetic Biology (Ministry of Education), Tianjin University, Tianjin, China; 7 Key Laboratory of Systems Bioengineering (Ministry of Education), Tianjin University, Tianjin, China; 8 Department of Chemistry, Tianjin University, Tianjin, China; University of Washington School of Medicine, Seattle, Washington, USA

**Keywords:** polyphenols degradation, anaerobic bacteria, carbon source, dihydrophloroglucinol cyclohydrolase

## Abstract

Polyphenols are abundant in nature, and their anaerobic biodegradation by gut and soil bacteria is a topic of great interest. The O_2_ requirement of phenol oxidases is thought to explain the microbial inertness of phenolic compounds in anoxic environments, such as peatlands, termed the enzyme latch hypothesis. A caveat of this model is that certain phenols are known to be degraded by strict anaerobic bacteria, although the biochemical basis for this process is incompletely understood. Here, we report the discovery and characterization of a gene cluster in the environmental bacterium *Clostridium scatologenes* for the degradation phloroglucinol (1,3,5-trihydroxybenzene), a key intermediate in the anaerobic degradation of flavonoids and tannins, which constitute the most abundant polyphenols in nature. The gene cluster encodes the key C-C cleavage enzyme dihydrophloroglucinol cyclohydrolase, as well as (*S*)-3-hydroxy-5-oxo-hexanoate dehydrogenase and triacetate acetoacetate-lyase, which enable phloroglucinol to be utilized as a carbon and energy source. Bioinformatics studies revealed the presence of this gene cluster in phylogenetically and metabolically diverse gut and environmental bacteria, with potential impacts on human health and carbon preservation in peat soils and other anaerobic environmental niches.

**IMPORTANCE:**

This study provides novel insights into the microbiota’s anaerobic metabolism of phloroglucinol, a critical intermediate in the degradation of polyphenols in plants. Elucidation of this anaerobic pathway reveals enzymatic mechanisms for the degradation of phloroglucinol into short-chain fatty acids and acetyl-CoA, which are used as a carbon and energy source for bacterium growth. Bioinformatics studies suggested the prevalence of this pathway in phylogenetically and metabolically diverse gut and environmental bacteria, with potential impacts on carbon preservation in peat soils and human gut health.

## INTRODUCTION

Polyphenols are structurally diverse metabolites that together constitute one of the most ubiquitous groups of plant natural products (1, 2). Polyphenols have historically been used in the tanning industry (1, 3). Their abundant production by plants, and subsequent degradation by microbes, makes polyphenols an important component of the biological carbon cycle. Certain anoxic biomes, such as peatlands and wetlands, accumulate large quantities of polyphenols and serve as important global carbon sinks (4, 5). The enzyme latch hypothesis suggests that the absence of O_2_-dependent phenolic degradation in anaerobic environments leads to the accumulation of polyphenols and consequent inhibition of microbial growth (4). However, recent studies of anoxic wetland soil microbiota demonstrated their ability to degrade diverse polyphenols, including condensed tannins, generally thought to be recalcitrant (5). Due to their antioxidant properties, polyphenols have also been used as nutritional supplements and are considered as health-promoting components in plant-derived foods, such as fruits, vegetables, legumes, nuts, and seeds (6). Dietary polyphenols are metabolized in the large intestine by microorganisms via processes such as deglycosylation, deconjugation, and dehydroxylation, modifying their bioactivity and absorptive properties (7). Polyphenol backbone cleavage has been attributed to several gut bacterial genera, including *Clostridium*, *Eubacterium*, *Flavonifractor,* and *Butyrivibrio*.

**Fig 1:**
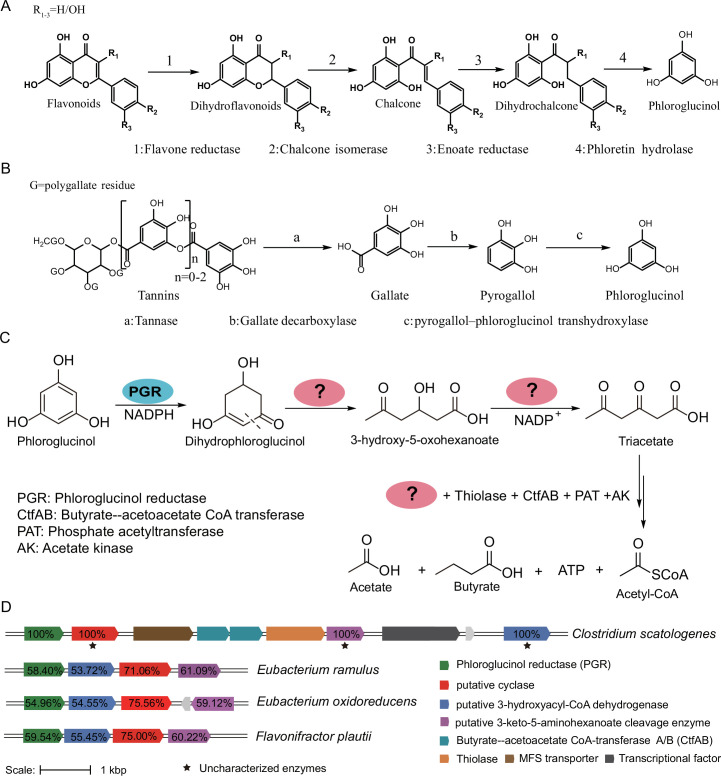
Phloroglucinol degradation as extension of polyphenol degradative pathways and its candidate gene clusters in anaerobic bacteria. (**A**) The degradation pathway of flavonoids involving formation of phloroglucinol (5, 8). (B) The degradation pathway of tannins involving formation of phloroglucinol (13, 15). (**C**) Proposed microbial metabolic pathway for phloroglucinol (17, 18, 22). The known enzyme PGR is labeled with a blue ellipse. Each of the unknown enzymes in the pathway is labeled with a question mark in a pink ellipse. (**D**) The putative gene cluster involved in phloroglucinol degradation in environmental and gut bacteria.

Anaerobic bacterial degradation of two of the most abundant classes of polyphenols, flavonoids (8), and tannic acids (5) involves phloroglucinol as a key intermediate. An FMNH_2_-dependent flavone reductase of the ene-reductase family in *Flavonifractor plautii* was discovered and characterized structurally and biochemically. It catalyzes the initial step of the flavonoid degradation pathway reducing flavonoids to form dihydroflavonoids and is found widespread in the gut microbiome (8). Following the formation of dihydroflavonoids, chalcone isomerase (9, 10), enoate reductase (9), and phloretin hydrolase (11, 12) act in order in the pathway to produce phloroglucinol (Fig. 1A). Tannins are degraded by sequential actions of the proposed tannase (13, 14), gallate decarboxylase (15 - 17), and Mo-dependent pyrogallol–phloroglucinol transhydroxylase (18 - 21) to form phloroglucinol as well (Fig. 1B).

The ability to mobilize the phenolic carbon for fermentative growth relies on further enzymatic degradation of phloroglucinol. A short-chain dehydrogenase and reductase family member, phloroglucinol reductase (PGR) dedicated to reduce phloroglucinol into dihydrophloroglucinol (DPG) using reducing equivalents from NADPH has been reported for both aerobic and anaerobic bacteria (22, 23). The recently reported crystal structure of PGR from *Clostridium* sp. ATCC BAA-442, and assays using [^2^H]-NADPH, supported a reaction mechanism involving the reduction of a keto tautomer of phloroglucinol (24). 3-Hydroxy-5-oxo-hexanoate and triacetate (3, 5-dioxo-hexanoate) were proposed to be downstream degradation products after DPG based on the study of the cell extracts of *Eubacterium oxidoreducens* (17) and *Pelobacter acidigallici* (18) (Fig. 1C). However, specific enzymes involved, including the key C-C cleavage enzyme proposed to catalyze dihydrophologlucinol retro-Claisen ring-opening reaction, have yet to be discovered. Identification of these enzymes could allow the prediction of polyphenol-degrading microbes from the exponentially growing genomic data in public sequence databases.

In this work, we report the bioinformatics discovery and biochemical and biophysical characterizations of a multi-enzyme system responsible for the degradation of phloroglucinol in *Clostridium scatologenes*, by which carbon and energy required to sustain bacterial growth are derived. This discovery completes the degradation pathway of flavonoids and adds to the enzymatic ring-opening mechanisms for aromatic compounds and energy metabolism in gut bacteria. Furthermore, with the identities of enzymes discovered, we were able to recognize other anaerobic bacteria, including the gut and environmental bacteria that are predicted to metabolize phloroglucinol.

## RESULTS

### Identification of candidate gene clusters involved in anaerobic phloroglucinol degradation

We noticed the genes known to encode enzymes involved in flavonoid degradation in *F. plautii* [the National Center for Biotechnology Information (NCBI) reference sequence: NZ_CP015406.2] and *E. ramulus* (NZ_JRFU00000000.1) are contained and similarly organized in the genome of *C. scatologenes* (NZ_CP009933.1), a well-studied malodorant-producing bacterium isolated from anaerobic sediments, capable of acetogenic and fermentative metabolism (25) (Supplementary Fig. 1). A putative PGR was also identified in this *C. scatologenes* strain (UniProt reference sequence: A0A0E3M5A2; denoted here as *Cs*PGR), which shares 59.54% sequence identity with the previously characterized PGR from *Clostridium* sp. ATCC BAA-442 (24). In search for genes involved in phloroglucinol degradation, we examined the neighborhood of *Cs*PGR and found an enzyme of unknown function of the “cyclase” family (A0A0E3JZE4), a transferase closely homologous to the 3-keto-5-aminohexanoate cleavage enzyme involved in lysine degradation (A0A0E3M5J5) and a putative 3-hydroxyacyl-CoA dehydrogenase (A0A0E3GQA2) in close proximity with *Cs*PGR (Fig. 1D) (17, 24). This gene cluster appears to be also conserved in *E. ramulus*, *E. oxidoreducens*, and *F. plautii*. In addition, the *C. scatologenes* gene cluster contains a major family transporter and transcriptional regulator, which are also found in conjunction with the putative PG degradation genes in other bacterial species.

### Validification of *Cs*PGR

**Fig 2:**
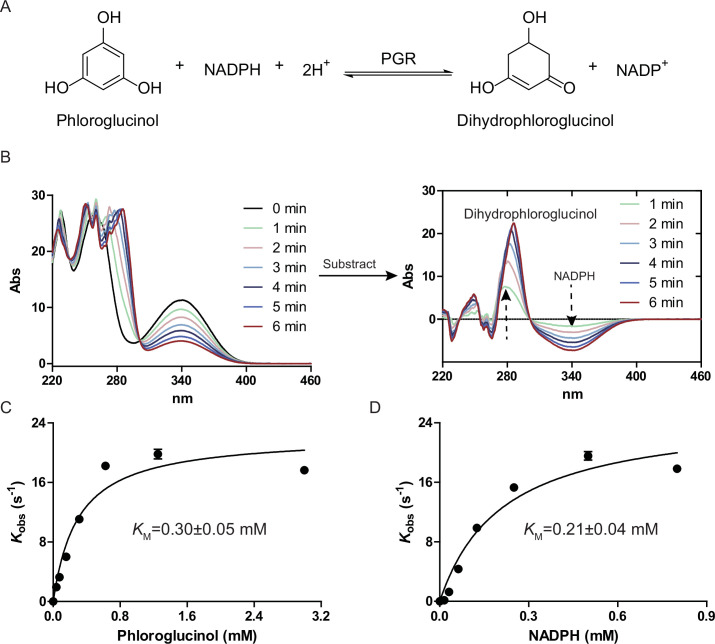
*Cs*PGR enzyme activity assays. (**A**) The PGR-catalyzed reaction. (**B**) Time-dependent UV-Vis spectra of *Cs*PGR assays. Inset: UV-Vis difference spectra with the UV-Vis spectrum at time 0 subtracted from each of the spectra collected at different time points. (**C**) Michaelis–Menten kinetics of *Cs*PGR varying the concentrations of phloroglucinol. (**D**) Michaelis–Menten kinetics of *Cs*PGR varying the concentrations of NADPH.

The gene *Cs*PGR was amplified by colony PCR and cloned into the pET28a by Gibson assembly. N-terminal His_6_-tagged *Cs*PGR was then overexpressed in *Escherichia coli* BL21(DE3) and purified to near homogeneity by Ni-NTA affinity chromatography (Supplementary Fig. 2). Incubation of phloroglucinol and NADPH with *Cs*PGR resulted in time-dependent changes in the absorption spectra, indicating a reaction occurring at the aromatic nucleus (Fig. 2A and B). Ultraviolet-visible (UV-Vis) difference spectra, obtained by subtracting the spectrum of the starting material from that of the product, exhibited a time-dependent increase in absorbance at 278 nm (Fig. 2B), consistent with the conversion of phloroglucinol into DPG (ε_278 nm_ = 29,986 M^−1^ cm^−1^) (23). During the reaction, a concomitant decrease in absorbance at 340 nm, indicating the consumption of NADPH (ε_340 nm_ = 6,220 M^−1^ cm^−1^) was also observed (Fig. 2B). The spectrophotometric assay monitoring NADPH oxidation was used to measure the enzyme kinetic parameters (*k*_cat_ = 22.4–25.3 s^−1^, *K_M_* equal to 0.30 and 0.21 mM for phloroglucinol and NADPH, respectively) (Fig. 2C and D). Structure-based sequence analysis of PGRs revealed a conserved TR(S/N) motif in the NADPH binding pocket interacting with its 2′-phosphate, contributing to NADP(H) substrate specificity over NAD(H) (Supplementary Fig. 3).

### Identification and characterization of an Mn^2+^-dependent dihydrophloroglucinol cyclohydrolyase in *C. scatologenes*

**Fig 3:**
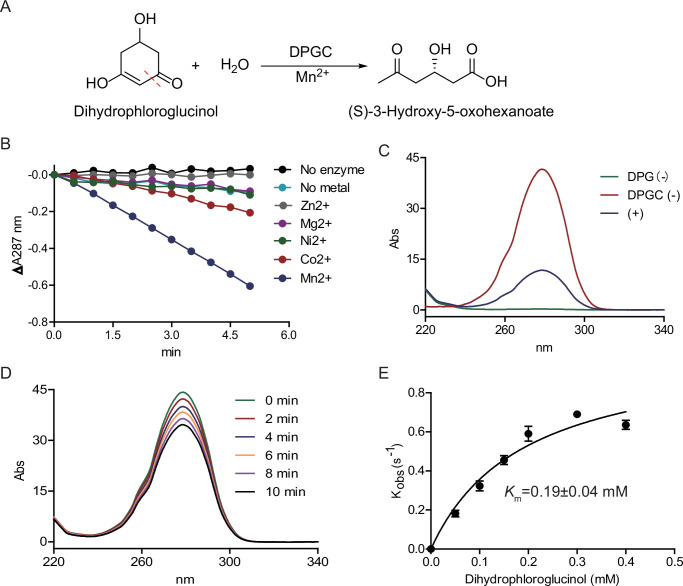
*Cs*DPGC enzyme activity assays. (**A**) The DPGC-catalyzed reaction. (**B**) Metal selectivity assays. (**C**) UV-Vis spectra of *Cs*DPGC assays. Negative controls omitted either DPG or *Cs*DPGC. (**D**) Time-dependent UV-Vis spectra of *Cs*DPGC assays. (**E**) Michaelis–Menten kinetics of *Cs*DPGC varying the concentrations of DPG.

The closest homolog that has been characterized of the “cyclase” enzyme (A0A0E3JZE4, putatively dihydrophloroglucinol cyclohydrolyase, *Cs*DPGC) in the gene cluster is an Mn^2+^-dependent isatin hydrolase (26) from *Labrenzia aggregata* with only 25% overall sequence identity (Supplementary Fig. 4A). Structure modeling and comparison showed that the metal binding sites of these two enzymes are highly conserved (Supplementary Fig. 4B and C). However, the essential residues involved in substrate recognition (Ile32 and Leu34) in isatin hydrolase are replaced by Trp20 and Tyr22 in *Cs*DPGC, respectively (Supplementary Fig. 4A and C), suggesting a different substrate. Analogies between the mechanism of the isatin amide C-N hydrolase reaction and that of the proposed DPG retro-Claisen “C-C hydrolase” reaction (Supplementary Fig. 4D) led us to hypothesize that *Cs*DPGC catalyzes the cleavage of DPG, forming 3-hydroxy-5-oxo-hexanoate (Fig. 3A). The “cyclase” family also includes DpsY, an enzyme proposed to catalyze C-C bond forming polyketide cyclization in the biosynthesis of daunorubicin (27).

We purified the recombinant *Cs*DPGC to homogeneity (Supplementary Fig. 2) and next performed enzyme activity assays with *Cs*DPGC as isolated or in the presence of additional Mn^2+^, or other divalent metals (Fig. 3B). Incubation of *Cs*DPGC with DPG resulted in time-dependent decrease in 278 nm (Fig. 3C and D). Without additional divalent metal, enzyme activity was barely detectable suggesting that the recombinant protein as purified was mostly in its apo form. Among all tested divalent metals, Mn^2+^ was optimal, and the enzyme retained about 25% activity when Co^2+^ was used as the cofactor (Fig. 3B). The enzyme activity of DPGC was inhibited by Zn^2+^ (Fig. 3B) similar to that of isatin hydrolase (26). The spectrophotometric assays varying substrate concentrations were carried out to measure enzyme kinetic parameters (*k*_cat_ = 1.04 ± 0.10 s^−1^, *K_M_* = 0.19 ± 0.04 mM) (Fig. 3E).

### Identification and characterization of an NAD(P)^+^-dependent (*S*)-3-hydroxy-5-oxohexanoate dehydrogenase in *C. scatologenes*

We next performed sequence and structure analyses of the as-annotated 3-hydroxyacyl-CoA dehydrogenase in the neighborhood of *C. scatologenes* genome (A0A0E3GQA2). And we proposed that it is indeed an NAD(P)^+^-dependent (*S*)-3-hydroxycarboxylate dehydrogenase [denoted here as *Cs*TfD for *C. scatologenes* triacetate-forming dehydrogenase (TfD)]. The mechanism of (*S*)-3-hydroxyacyl-CoA dehydrogenase has been extensively investigated, both biochemically and structurally (28 - 30). Compared with the (*S*)-3-hydroxybutyryl-CoA dehydrogenase from *C. butyricum* (29) and *C. acetobutylicum* (30), *Cs*TfD lacks conserved Lys56 and Asn221, which are involved in hydrogen bond interactions with 2′-phosphate moiety and pantothenic moiety of the substrate, respectively (29, 30) (Supplementary Fig. 5A and C). However, the essential residues involved in catalysis (His138) and substrate stereo-specificity recognition (Ser117 and Asn188) in (*S*)-3-hydroxybutyryl-CoA dehydrogenase are highly conserved in *Cs*TfD (His143, Ser122, and Asn194) (Supplementary Fig. 5A and C).

**Fig 4:**
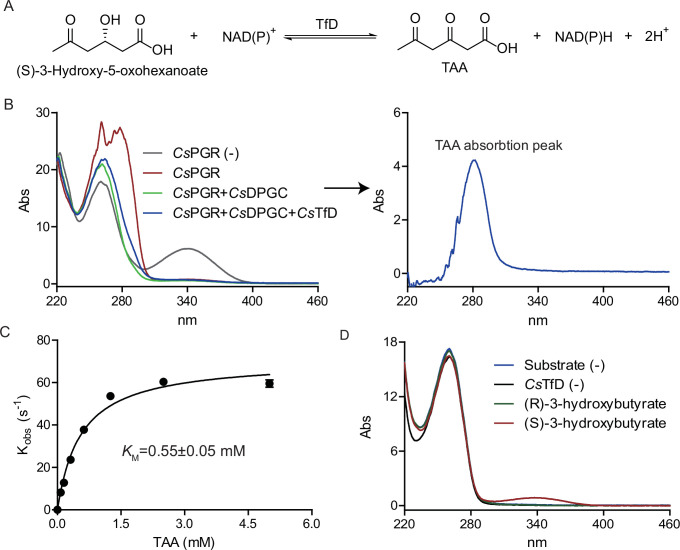
*Cs*TfD enzyme activity assays. (**A**) The TfD-catalyzed reaction. (**B**) Time-dependent UV-Vis spectra of *Cs*TfD assays. Inset: TAA formation upon subtracting the spectrum of the *Cs*PGR-*Cs*DPGC reaction mixture from the spectrum of the *Cs*PGR-*Cs*DPGC-*Cs*TfD reaction mixture. (**C**) Michaelis–Menten kinetics of *Cs*TfD varying the concentrations of TAA. (**D**) The configuration selectivity of *Cs*TfD substrates.

*Cs*TfD was purified to near homogeneity by Ni-NTA affinity chromatography (Supplementary Fig. 2). The substrates of *Cs*TfD, both 3-hydroxy-5-oxohexanoate and NADP^+^, can be provided by the *Cs*PGR-*Cs*DPGC coupling enzyme reaction. Incubation of *Cs*TfD with *Cs*PGR-*Cs*DPGC reaction mixtures resulted in changes in the absorption spectra (Fig. 4A and B), UV-Vis difference spectra, obtained by subtracting the spectrum of the starting material (*Cs*PGR-*Cs*DPGC reaction mixtures) from that of the product, exhibited a maximum (λmax) at 281 nm (Fig. 4B) consistent with the conversion of 3-hydroxy-5-oxohexanoate into a triacetate (λmax, 276 nm [31]). Incubation of *Cs*TfD with saturating amounts of TAA (triacetate) and NAD(P)H resulted in time-dependent decrease in 340 nm [NAD(P)H ε_340 nm_ = 6,220 M^−1^ cm^−1^] (Supplementary Fig. 6A through D). The spectrophotometric assay monitoring NAD(P)H oxidation was used to measure the kinetic parameters of the substrates. The *k*_cat_ ranges from 78.5 to 395.0 s^−1^, depending on the choice of the nucleotide reductant. The *K_M_* was determined to be 0.55 mM for TAA (Fig. 4C), and the *K_M_* for NADH was determined to be 0.13 mM (Supplementary Fig. 6D) much lower than that for NADPH, which could only be estimated to be 1.7 mM (Supplementary Fig. 6B). The enzyme has a much lower activity for NADPH-dependent reduction of acetoacetate (0.02% relative to TAA) (Supplementary Fig. 6E), and the *K_M_* is 3.69 mM for acetoacetate (Supplementary Fig. 6F). *Cs*TfD favors (*S*)-3-hydroxybutyrate over (*R*)-3-hydroxybutyrate as its substrate for the reverse oxidation reaction (Fig. 4D), suggesting that the physiological substrate of *Cs*TfD and the product of *Cs*DPGC, 3-hydroxy-5-oxohexanoate should be in its (*S*)-configuration. Therefore, *Cs*TfD is an NAD(P)^+^-dependent (*S*)-3-hydroxy-5-oxo-hexanoate dehydrogenase.

### Identification and characterization of a triacetate acetoacetate-lyase in *C. scatologenes*

**Fig 5:**
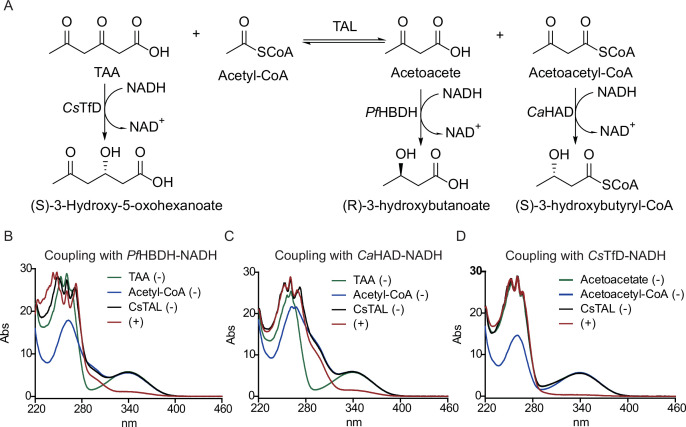
*Cs*TAL enzyme activity assays. (**A**) The TAL-catalyzed reaction and coupling enzyme reactions used for activity assays from (B) to (D). (**B**) UV-Vis spectra of *Cs*TAL activity assays in couple with *Pf*HBDH (33). Negative controls omitted triacetate, acetyl-CoA, or *Cs*TAL. (**C**) UV-Vis spectra of *Cs*TAL activity assays in couple with *Ca*HAD (34). Negative controls omitted triacetate, acetyl-CoA, or TAL. (**D**) UV-Vis spectra of *Cs*TAL activity assays in couple with *Cs*TfD. Negative controls omitted acetoacetate, acetoacetyl-CoA, or *Cs*TAL.

Sequence and structure analyses suggested that the as-annotated 3-keto-5-aminohexanoate cleavage enzyme in *C. scatologenes* (A0A0E3M5J5, denoted here as *Cs*TAL) could have been a Zn^2+^-dependent triacetate acetoacetate-lyase (TAL). Compared with the structure of 3-keto-5-aminohexanoate cleavage enzyme (2Y7F [32]), most of the residues in the substrate binding pocket of *Cs*TAL are highly conserved (Supplementary Fig. 7). Strikingly, an exception is noted for Glu14 which is required to interact with the 5-amino group of 3-keto-5-aminohexanoate and is replaced by Trp16 in *Cs*TAL (Supplementary Fig. 7A and C). This substitution is consistent with the hypothesis of the reaction substrate being 3,5-dioxohexanoate, forming acetoacetate and acetoacetyl-CoA (Fig. 5A). To test this hypothesis, *Cs*TAL was purified to near homogeneity by Ni-NTA affinity chromatography (Supplementary Fig. 2). Incubation of *Cs*TAL with triacetate and acetyl-CoA resulted in the formation of acetoacetate and acetoacetyl-CoA, which can be monitored at 340 nm due to the consumption of NADH in coupling enzyme reactions with D-3-hydroxybutyrate dehydrogenase from *Pseudomonas fragi* (33) and (*S*)-3-hydroxybutyryl-CoA dehydrogenase from *C. acetobutylicum* (34), respectively (Fig. 5A through C). The *Cs*TAL enzyme activity catalyzing the reverse reaction with acetoacetate and acetoacetyl-CoA as substrates was also confirmed in couple with *Cs*TfD (Fig. 5A and D).

### Reconstitution of the phloroglucinol degradation pathway

*In vitro* reconstitution of the phloroglucinol degradation pathway was carried out with purified recombinant *C. scatologenes* enzymes (PGR, DPGC, TfD, and TAL) and phloroglucinol, NADPH, and acetyl-CoA as substrates. The reaction mixture and controls were analyzed by LC-MS (liquid chromatography–mass spectrometry), with separation carried out on a C18 column, and detection by ESI-MS (electrospray ionization mass spectrometry) in the negative ionization mode. Incubation of phloroglucinol (*m*/z = 125) (Supplementary Fig. 8A) and NADPH with *Cs*PGR led to a decrease of the phloroglucinol peak, and the appearance of a new peak corresponding to DPG (*m*/z = 127) (Supplementary Fig. 8B), consistent with the NADPH-dependent reduction of phloroglucinol to DPG. Incubation of phloroglucinol and NADPH with *Cs*PGR and *Cs*DPGC led to a decrease in the DPG peak, and the appearance of a new peak corresponding to (*S*)-3-hydroxy-5-oxohexanoate (*m*/z = 145) (Supplementary Fig. 8C), suggesting that *Cs*DPGC catalyzes the ring-opening C-C bond cleavage of DPG. Incubation of phloroglucinol and NADPH with *Cs*PGR, *Cs*DPGC, and *Cs*TfD results in the decrease of the (*S*)-3-hydroxy-5-oxo-hexanoate peak, and appearance of a new peak corresponding to triacetate (*m*/z = 143), consistent with the NADP^+^-dependent oxidation of (*S*)-3-hydroxy-5-oxohexanoate to form triacetate by *Cs*TfD (Supplementary Fig. 8D). Incubation of phloroglucinol, NADPH, and acetyl-CoA with all four enzymes *Cs*PGR, *Cs*DPGC, *Cs*TfD, and *Cs*TAL led to the disappearance of the triacetate peak and appearance of a new peak corresponding to acetoacetate (*m*/z = 101) (Supplementary Fig. 8E).

**Fig 6:**
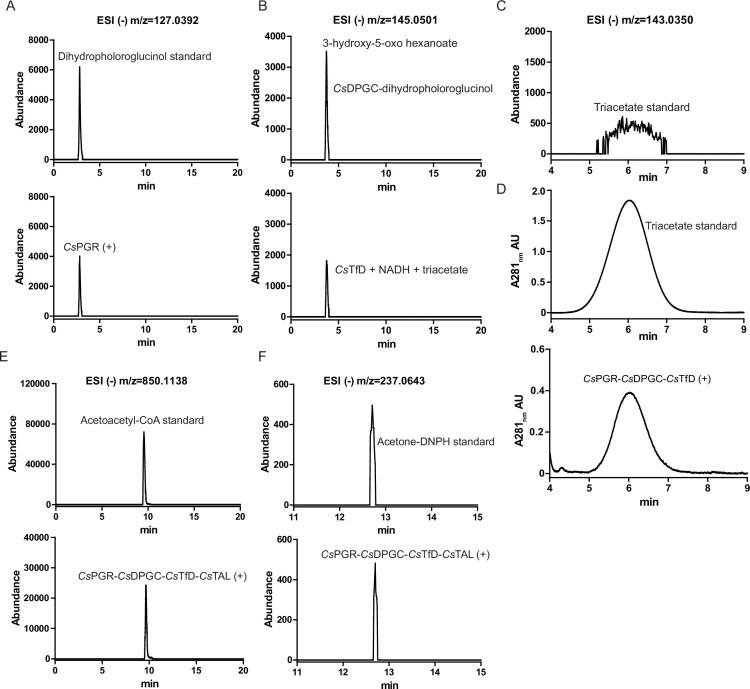
The major products of enzymatic reactions were characterized by high-resolution LC-UV/MS. (**A**) LC-MS elution profile of dihydrophologlucinol and *Cs*PGR reaction products. (**B**) LC-MS elution profile of *Cs*DPGC reaction products and *Cs*TfD reaction products. (**C**) LC-MS elution profile of triacetate. (**D**) LC-UV elution profile of triacetate and *Cs*PGR-*Cs*DPGC-*Cs*TfD reaction products. (**E**) LC-MS elution profile of acetoacetyl-CoA and *Cs*PGR-*Cs*DPGC-*Cs*TfD-*Cs*TAL reaction products. (**F**) LC-MS elution profile of acetone-DNPH and *Cs*PGR-*Cs*DPGC-*Cs*TfD-*Cs*TAL reaction products.

**Fig 7:**
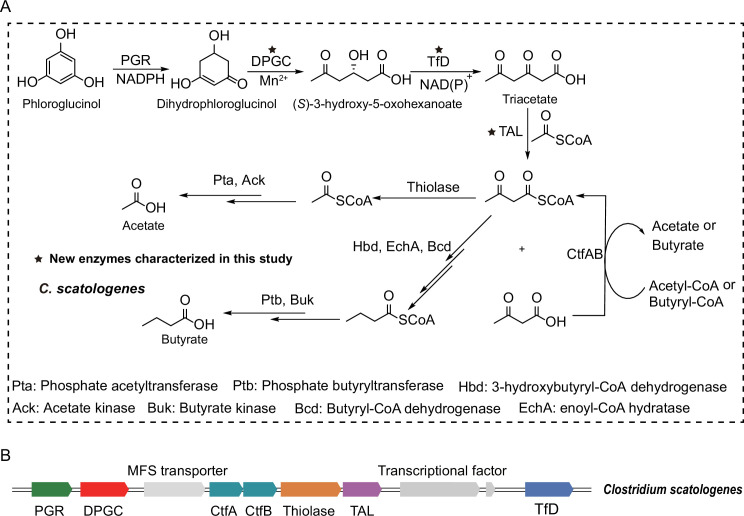
Phloroglucinol supports the growth of *C. scatologenes* as a carbon and energy source. (**A**) Proposed metabolic pathways with phloroglucinol as a carbon and energy source for *C. scatologenes*. (**B**) The re-annotated *C. scatologenes* gene cluster involved in phloroglucinol metabolism.

Further experiments were conducted to ascertain the identity of the enzymatic reaction products, including dihydrophologlucinol, 3-hydroxy-5-oxo-hexanoate, triacetate, acetoacetyl-CoA, and acetoacetate. Dihydrophloroglucinol, triacetate, and acetoacetyl-CoA were analyzed by high-resolution LC-UV/MS, showing co-elution with commercial standards (Fig. 6A and C through E; Supplementary Fig. 9). 3-Hydroxy-5-oxo-hexanoate, formed by both dihydrophologlucinol hydrolysis catalyzed by DPGC and triacetate reduction catalyzed by TfD, was also analyzed by high-resolution LC-MS and showed co-elution of the two reaction products (Fig. 6B; Supplementary Fig. 9). Acetoacetate, formed from the sequential reactions of DPGC, TfD, and TAL, could not be detected by high-resolution LC-MS, likely due to decarboxylation to form acetone during the workup and ionization processes. However, derivatization with 2,4-dinitrophenylhydrazine (DNPH) followed by high-resolution LC-MS analysis demonstrated the formation of DNPH-acetone, showing co-elution with a commercial standard (Fig. 6F; Supplementary Fig. 9). The LC-MS data are consistent with the proposed phloroglucinol degradation pathway (Fig. 7A).

### Phloroglucinol was used as a carbon and energy source for *C. scatologenes* growth

Next, we decided to investigate the physiological role of the gene cluster in utilizing phloroglucinol as a carbon and energy source. *C. scatologenes* was anaerobically grown in a defined medium with glucose or phloroglucinol as a carbon source. The *C. scatologenes* cells grew robustly when glucose was provided, and the culture turned turbid with the optical density at 600 nm reaching 1.4 within 2–3 d. By contrast, cells grew much slower when phloroglucinol was provided as a carbon source, the OD_600_ reached 0.32 after prolonged incubation at 37°C for 7–8 d (Supplementary Fig. 10A). The OD_600_ of the negative control without carbon source was less than 0.08. Sodium dodecyl sulfate polyacrylamide gel electrophoresis (SDS/PAGE) analysis revealed multiple prominent protein bands migrating in the region from 20 to 45 kDa, present in phloroglucinol-, but not glucose-grown cells. Mass spectrometric analyses identified the prominent protein bands being *Cs*PGR, DPGC, TfD, TAL, thiolase, acetate kinase, and butyrate kinase (Fig. 7B; Supplementary Fig. 10B; Supplementary data 1), suggesting that induction of the gene cluster occurred in response to the presence of phloroglucinol as a carbon source in the growth medium. Growth on the phloroglucinol medium was accompanied by formation of acetate and butyrate, as detected by LC-MS (Supplementary Fig. 11). The transcript levels of PGR, DPGC, TfD, and TAL genes in *C. scatologenes* were investigated using reverse transcription quantitative polymerase chain reaction (qPCR; Table S2; Supplementary Fig. 12). Cells grown on phloroglucinol as the sole carbon source were insufficient for RNA isolation; therefore, cells were grown on glucose and phloroglucinol instead. Compared with growth on glucose alone, growth on glucose and phloroglucinol led to a 4.2- to 5.7-fold induction of the four genes.

### Presence of the phloroglucinol degradation pathway in diverse bacteria

**Fig 8:**
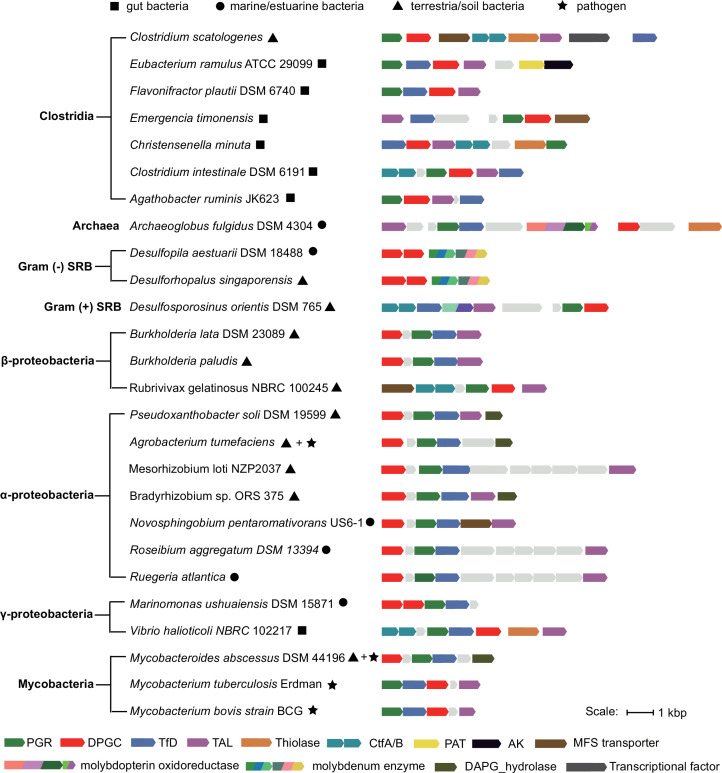
Presence of the phloroglucinol degradation pathway in phylogenetically and metabolically diverse bacteria. AK, acetate kinase; CtfAB, acetoacetyl-CoA:acetate/butyrate CoA transferase; PAT, phosphate acetyltransferase. DAPG_hydrolase, phloretin hydrolase. Circle, gut bacteria; square, marine/estuarine bacteria; triangle, soil/terrestrial bacteria; star, pathogen.

To examine the prevalence of this pathway, we employed cblaster (version 1.3.16) (35) to search for co-located homologs of *C. scatologenes* PGR, DPGC, TfD, and TAL in NCBI genome database (12,225 gene cluster, Supplementary data 2). To facilitate further analysis using UniProt-based tools (36), we used the UniProt ID mapping tool to map the DPGC RefSeq accession numbers from the cblaster output onto the corresponding UniProt UniRef50 cluster numbers (six UniRef50 clusters containing 524 UniProt entries; Supplementary data 3). The genome neighborhoods of the DPGC homologs were examined using the Enzyme Function Initiative Genome Neighborhood Tool (37), revealing the presence of gene clusters encoding this phloroglucinol degradation pathway in phylogenetically and metabolically diverse bacteria from different environments (Fig. 8; Supplementary data 4).

The gene cluster was present in several bacteria that are reported to degrade phloroglucinol, including strict anaerobic Gram-positive butyrate-producing fermenting bacteria from the environment and digestive system of humans and herbivorous animals (*E. ramulus*, *E. oxidoreducens*, *F. plautii*, and *C. scatologene*s reported here), and the facultative anaerobic phototrophic Betaproteobacterium *Rubrivivax gelatinosus* isolated from food wastewater (38). In addition, the gene cluster was also present in organisms that have not previously been reported to degrade phloroglucinol, including marine alpha- and gamma-proteobacteria, anaerobic Gram-negative and Gram-positive sulfate-reducing bacteria, and sulfate-reducing archaea belonging to the genus Archaeoglobus, which may play roles in polyphenol mineralization in marine, coastal, and estuarine sediments. Presence of the gene cluster in *Mesorhizobium loti* and *Bradyrhizobium* sp., which were previously reported to degrade flavonoids to produce phloroglucinol, suggests that certain strains of these soil bacteria and plant symbionts or pathogens may be able to further degrade phloroglucinol. Presence of the gene cluster in various *Mycobacterium* species, which are inhabitants of soil and water and are also human and animal pathogens, suggests an ability to degrade polyphenols. Notably, the gene cluster in *Mycobacterium* species also contains phloretin hydrolase, which was previously reported in *Mycobacterium abscessus* (39). Collectively, these observations suggest a central role for this phloroglucinol degradation pathway in mobilization of phenolic carbons by diverse bacteria in different environments.

## DISCUSSION

Through a combination of bioinformatics, biochemical, and biophysical studies, we have identified and characterized the enzymes involved in the anaerobic phloroglucinol degradation pathway in *C. scatologenes*. In this pathway, the key retro-Claisen C-C cleavage of DPG to (*S*)-3-hydroxy-5-oxo-hexanoate is catalyzed by DPGC, a member of the “cyclase” family. NAD(P)-dependent oxidation of (*S*)-3-hydroxy-5-oxo-hexanoate is catalyzed by a stereospecific NAD(P)^+^-dependent dehydrogenase to form triacetate, followed by the second C-C cleavage catalyzed by TAL, a member of the “beta-keto acid cleavage enzyme” family (40). The gene cluster also contains a homolog of thiolase, which catalyzes the conversion of acetoacetyl-CoA into acetyl-CoA, and acetyl/butyryl-CoA:acetoacetyl-CoA transferase, which converts acetoacetate to acetoacetyl-CoA. Acetoacetyl-CoA and acetyl-CoA are intermediates in the butyrate fermentation pathway, allowing for the production of ATP via phosphate acetyl/butyryl-transferase and acetate/butyrate kinase and further energy conservation via crotonyl-CoA reduction (Fig. 7A).

Previously, the anaerobic phloroglucinol degradation has been studied in the Gram-positive bacterium *E. oxidoreducens* (17) and the Gram-negative bacterium *P. acidigallici* (whose genome is currently not available) (18). The degradation process is believed to proceed through similar pathways in both bacterial strains. However, while *P. acidigallici* degrades phloroglucinol to produce a stoichiometric amount of acetate, *E. oxidoreducens* requires an external electron donor, such as H_2_ or formate, to grow on phloroglucinol and generates both acetate and butyrate. The differences in the need for an external electron donor are attributed to the divergent mechanisms employed by the two bacterial strains to regenerate NADPH, a requisite for phloroglucinol reduction. Specifically, while the oxidation of 3-hydroxy-5-oxo-hexanoate in *P. acidigallici* produces NADPH, the oxidation of the 3-hydroxyacid or 3-hydroxyacyl-CoA intermediate in *E. oxidoreducens* is thought to generate NADH, necessitating NADPH generation via hydrogenase or formate dehydrogenase. Our experiments with recombinant *C. scatologenes* TfD demonstrate its preference for NADH over NADPH and that growth on phloroglucinol generates both acetate and butyrate, similar to *E. oxidoreducens*. In our experiments, growth of *C. scatologenes* required the inclusion of amino acids in its growth medium, which could function as the external electron donor instead of H_2_ or formate.

Besides the anaerobic phloroglucinol degradation pathway described above, there exist several other known mechanisms for affecting anaerobic phenolic ring cleavage (41). These pathways typically involve either reductive or oxidative dearomatization, followed by ring cleavage via a retro-Claisen reaction. Degradation of phenol, catechol, and hydroquinone involves the formation of a benzoyl-CoA intermediate (41), which undergoes reductive dearomatization catalyzed by benzoyl-CoA reductase, followed by hydrolytic ring cleavage. In some bacteria, resorcinol degradation takes place via reductive dearomatization catalyzed by resorcinol reductase (42), followed by hydrolytic ring cleavage. Alternatively, in other bacteria, resorcinol degradation proceeds through hydroxylation to form hydroxyhydroquinone (43), followed by oxidative dearomatization, and then hydrolytic cleavage. The degradation of pyrogallol involves its conversion into phloroglucinol by the molybdoenzyme pyrogallol–phloroglucinol transhydroxylase, followed by degradation through the anaerobic phloroglucinol degradation pathway. Phloroglucinol and resorcinol have hydroxyl groups that are located in the meta position with respect to each other. As a result, their keto tautomers (1,3,5-trioxocyclohexane and 1,3-dioxocyclohexene) are more stable, which could explain why they can be reduced relatively easily by the NADPH-dependent PGR and the ferredoxin-dependent resorcinol reductase, respectively (44, 45). By contrast, the reduction of benzoyl-CoA is believed to follow a mechanism similar to Birch reduction, requiring a much stronger reductant. In class I benzoyl-CoA reductase, this reduction is driven by stoichiometric ATP hydrolysis, while in class II benzoyl-CoA reductase, it is driven by electron bifurcation (45).

Our bioinformatics analysis revealed the presence of gene clusters encoding the phloroglucinol degradation pathway in metabolically diverse bacteria, including strict anaerobic Gram-positive fermenting bacteria, strict anaerobic sulfate-reducing bacteria and archaea, and facultative anaerobic Gram-negative bacteria. These organisms inhabit diverse environmental niches (Fig. 8; Supplementary data 4), and some are plant or animal commensals or pathogens, suggesting that microbial phloroglucinol degradation ability is widespread. Identification of these gene clusters will facilitate further investigation of the fate of phenolic carbon in different ecologically important biomes.

The findings described here are also of great relevance to polyphenol biochemistry in the human intestinal microbiome. Studies of gut microbial polyphenol metabolism have focused on the production of bioactive polyphenol derivatives, such as the conversion of isoflavones to equol (46) and ellagitannins to urolithin (47). These involve reduction, dehydroxylation, and decarboxylation reactions, but not phenolic ring opening. By contrast, the phloroglucinol degradation pathway allows for the mobilization of phenolic carbons for fermentative energy metabolism, which is particularly important for the microbiota inhabiting the nutrient-scarce distal gut. Moreover, elucidation of this pathway reveals enzymatic mechanisms for the conversion of polyphenols into short-chain fatty acids (acetate and butyrate), important mediators of gut health (48, 49), providing another route through which microbial polyphenol metabolism can influence human health.

## MATERIALS AND METHODS

### Materials and general methods

Lysogeny broth (LB) was purchased from Sangon Biotech (Shanghai, China). Ultrapure deionized water from Millipore Direct-Q was used in this work. HIS*BIND RESIN (69670—10 mL) was purchased from EMD Millipore Corp. (USA). All protein purification chromatographic experiments were performed on gravity columns. Phloroglucinol dihydrate was purchased from Aladdin (Shanghai, China). DPG was synthesized by chemical reduction of phloroglucinol with NaHB_4_ according to the method of Patel et al. (23). The purity and identity of DPG were confirmed by UV spectroscopic and LC-MS analyses. Methyl 3,5-dioxohexanoate was purchased from Bidepharm (Shanghai, China), and triacetate was obtained by the ester hydrolysis of methyl 3,5-dioxohexanoate with 5N LiOH in 75% methanol system for 5 h. Other chemicals unless otherwise specified, including acetyl-CoA (A281-10MG) and acetoacetyl-CoA (A1625-5MG) and NAD(P)(H), were purchased from Sigma-Aldrich. UV-Vis spectroscopic measurements were monitored using a Biotek synergy 2 reader for 96-well plates and using a NANODROP ONE (Thermo Fisher Scientific, MA, USA ) otherwise. Kinetic parameters for the enzyme assays were extracted using GraphPad Prism 5.0.

### Multiple sequence alignment of PGRs, DPGCs, TfDs, and TALs

Clustal Omega (50) was used to generate multiple sequence alignments for selected representative sequences of PGRs, DPGCs, TfDs, and TALs from *C. scatologenes*, *E. ramulus*, and *F. plautii* and their homologous proteins, respectively.

### Homology modeling of *Cs*DPGC, *Cs*TfD, *Cs*TAL, and molecular docking

The models of *Cs*DPGC (A0A0E3JZE4), *Cs*TfD (A0A0E3GQA2), and *Cs*TAL (A0A0E3M5J5) were downloaded from AlphaFold DB (51, 52) (https://alphafold.ebi.ac.uk) and used for molecular docking and structural analysis. Induce-Fit Docking in Schrödinger Suite 2019-1 was employed for substrate docking.

### Gene cloning and syntheses

The genes encoding *Cs*PGR (NCBI reference sequence: AKA68207.1), *Cs*DPGC (AKA68208.1), and *Cs*TAL (AKA68213.1) were amplified by colony PCRs with the primers listed in Table S1, while the *Cs*TfD (AKA68216.1) gene was codon optimized and synthesized by General Biol Inc. (Anhui, China). These genes were then inserted by Gibson assembly (53) into the pET-28a (+) vector (54) for expression of N-terminal His_6_-tagged recombinant proteins in *E. coli*.

### Expression and purification of *Cs*PGR, *Cs*DPGC, *Cs*TfD, and *Cs*TAL

*E. coli* BL21 (DE3) cells were transformed with the pET-28a (+) plasmids encoding *Cs*PGR, *Cs*DPGC, *Cs*TfD, and *Cs*TAL genes and plated on LB agar supplemented with 50 µg/mL kanamycin. Transformants were grown in the LB medium (200–300 mL) at 37°C in a shaking incubator at 200 rpm. When OD_600_ reached 0.6–0.8, the temperature was decreased to 18°C, and 0.3 mM isopropyl β-D-1-thiogalactopyranoside was added to induce the production of the protein of interest. After 16–20 h, cells were harvested by centrifugation (6,000 × *g* for 10 min at 4°C). Cells (*Cs*PGR, *Cs*TfD, and *Cs*TAL) were resuspended in 20 mL of lysis buffer [50 mM Tris-HCl, pH 8.0, 1 mM phenylmethanesulfonyl fluoride (PMSF), 0.2 mg/mL lysozyme, 0.03% Triton X-100, and 0.02 mg/mL DNase I]. The cell suspension was frozen in a −80°C freezer and then thawed and incubated in a 25°C water bath for 30 min to allow for cell lysis. The cells containing *Cs*DPGC were resuspended in 20 mL of lysis buffer (20 mM Tris-HCl, pH 7.5, 200 mM KCl, 0.03% Triton X-100, and 1 mM PMSF) and were ruptured by probe sonication. About 2 mL of 11% streptomycin sulfate (dissolved in water) was added to the cell lysate followed by gentle mixing and centrifugation (20,000 × *g* for 10 min at 4°C). The supernatant was filtered through a 0.22-µm filter and loaded onto a 2-mL Ni-NTA affinity column pre-equilibrated with buffer A [20 mM Tris-HCl, pH 7.5, 5 mM β-mercaptoethanol, and 0.2 M KCl]. The column was washed with 10 column volumes (CVs) of buffer A and then 5 CVs of buffer A containing 40 mM imidazole, finally the protein was eluted with 5 CVs of buffer A containing 250 mM imidazole. The eluate of proteins was dialyzed or concentrated by the centrifuge concentrator (10K MWCO [molecular weight cutoff]; Millipore) for buffer exchange to remove imidazole. The concentrations of purified proteins were calculated from their absorptions at 280 nm. The purified proteins were examined on a 10% SDS polyacrylamide gradient gel and visualized by Coomassie staining.

### UV-Vis spectrophotometric assays of *Cs*PGR

A 40-µL reaction mixture, containing 50 mM Tris-HCl, pH 7.5, 2 mM phloroglucinol, 2 mM NAD(P)H, and 1 µM of *Cs*PGR was incubated for 0–6 min at room temperature (RT). The absorbance from 190 to 850 nm was monitored. To measure the Michaelis–Menten kinetic constants, the absorbance at 340 nm of a 200-µL reaction mixture, containing 50 mM Tris-HCl, pH 7.5, 0.05 µM of *Cs*PGR, 0–3.0 mM of phloroglucinol, and 0–0.8 mM NADPH in a 96-well plate, was monitored at 15 s intervals for 2 min at RT. Δ*A*_340 nm_ and the extinction coefficient of NADPH (6,220 M^−1^ cm^−1^) were used to calculate the reaction rates.

### The metal selectivity for *Cs*DPGC

A 200-µL reaction mixture, containing 50 mM Tris-HCl, pH 7.5, 0.2 mM DPG, 2 mM Mn^2+^ (or other divalent metal ions, ZnSO_4_·7H_2_O, MgCl_2_·6H_2_O, Ni_2_SO_4_, CoCl_2_, or MnCl_2_·4H_2_O), and 0.2 µM of *Cs*DPGC in a UV transparent 96-well plate (Corning 3635), was monitored at 278 nm at 30 s intervals for 5 min at RT. Δ*A*_278 nm_ and the extinction coefficient of DPG (29,968 M^−1^ cm^−1^ [23]) were used to calculate the reaction rates.

### UV-Vis spectrophotometric assays of *Cs*DPGC

A 40-µL reaction mixture, containing 50 mM Tris-HCl, pH 7.5, 1.5 mM DPG, 2 mM Mn^2+^, and 3 µM of *Cs*DPGC, was incubated for 0–20 min at RT. The absorbance from 220 to 350 nm was monitored. To measure the Michaelis–Menten kinetic constants, the absorbance at 278 nm of a 100-µL reaction mixture, containing 50 mM Tris-HCl, pH 7.5, 0.2 µM of *Cs*DPGC, 0–0.4 mM of DPG, and 2 mM Mn^2+^ in a UV transparent 96-well plate (Corning 3635), was monitored at 30 s intervals for 5 min at RT. Δ*A*_278 nm_ and the extinction coefficient of DPG (29,968 M^−1^ cm^−1^) were used to calculate the reaction rates.

### UV-Vis spectroscopic assays of the *Cs*PGR-*Cs*DPGC-*Cs*TfD coupling reaction

A 40-µL reaction mixture, containing 50 mM Tris-HCl, pH 7.5, 2 mM phloroglucinol, 2 mM NADPH, 2 µM of *Cs*PGR, 2 µM of *Cs*DPGC, 2 mM Mn^2+^, and 2 µM of *Cs*TfD, was incubated for 30 min at RT. The absorbance from 190 to 850 nm was monitored.

### UV-Vis spectroscopic assays of *Cs*TfD

In a typical end point assay, a 40-µL reaction mixture, containing 50 mM Tris-HCl, pH 7.5, 1 mM triacetate, 1 mM NADPH, and 0.1 µM of *Cs*TfD, was incubated for 10 min at RT, followed by UV-Vis absorbance scan from 190 to 850 nm. A 10-fold less *Cs*TfD (10 nM) was used when NADPH was replaced by NADH as the reductant. To measure the Michaelis–Menten kinetic constants, the concentration of *Cs*TfD was varied from 5 to 300 nM, the concentration of the substrate was varied in the range of 0–1.0 mM for NADPH or 0–0.75 mM for NADH, in the presence of a saturating concentration of 2.0 mM triacetate. In another set of experiments, the concentration of NADPH was fixed at 1.0 mM, while the triacetate concentration was varied from 0 to 5 mM, or acetoacetate concentration was varied from 0 to 50 mM. The reaction system was monitored at 5–60 s intervals for 1–10 min at RT. Δ*A*_340 nm_ and the extinction coefficient of NAD(P)H (6,220 M^−1^ cm^−1^) were used to calculate the reaction rates.

### UV-Vis spectroscopic assays of *Cs*TAL

To detect formation of the acetoacetate product, a coupling enzyme (*R*)-3-hydroxybutyrate dehydrogenase from *P. fragi* (33) (*Pf*HBDH: S31008-1KU, Shanghai Yuanye Bio-Technology Co., Ltd) was used. A 40-µL reaction mixture, containing 50 mM Tris-HCl, pH 7.5, 2 mM triacetate, 1 mM acetyl-CoA, 5 µM of *Cs*TAL, 0.5 U *Pf*HBDH, and 1 mM NADH, was incubated for 10 min at RT. To detect formation of the acetoacetyl-CoA product, a coupling enzyme (*S*)-3-hydroxybutyryl-CoA dehydrogenase from *C. acetobutylicum* (34) (*Ca*HAD) was used. A 40-µL reaction mixture, containing 50 mM Tris-HCl, pH 7.5, 4 mM triacetate, 1 mM acetyl-CoA, 5 µM of *Cs*TAL, 1 µM of *Ca*HAD, and 1 mM NADH, was incubated for 10 min at RT. To detect formation of triacetate as a product of the reversible reaction, a coupling enzyme *Cs*TfD was used. A 40-µL reaction mixture, containing 50 mM Tris-HCl, pH 7.5, 4 mM acetoacetate, 1 mM acetoacetyl-CoA, 5 µM of *Cs*TAL, 1 µM *Cs*TfD, and 1 mM NADH, was incubated for 10 min at RT. The absorbance spectra (190–850 nm) of the reaction mixtures at the end of the assays were collected.

### LC-MS analyses of the *Cs*PGR-*Cs*DPGC-*Cs*TfD-*Cs*TAL coupling reactions

An amount of 2 mM phloroglucinol, 1 mM NADPH, 2 mM Mn^2+^, and 0.5 mM acetyl-CoA in 300 µL reaction buffer (20 mM Tris-HCl, pH 7.5) was mixed and incubated with 2 µM of the enzyme cocktail for 1 h at RT. At the end of the assay, the reaction mixture was applied to a centrifuge concentrator (3K MWCO; Millipore). The flow-through was analyzed using an Agilent 6460 Triple Quadrupole LC/MS instrument (Agilent Technologies, Santa Clara, CA, USA). The LC separation was performed on Agilent ZORBAX Eclipse XDB-C18 (150 × 2.1 mm, product number 993700–902) column with a flow rate of 0.3 mL/min at RT. 0.1% Formate in water (solvent A) and acetonitrile (solvent B) was employed as mobile phase. A gradient of 0–8 min 0%−5% B, 8–9 min 5%−100% B, and 9–10 min 100% B was used. The sample injection volume was 5 µL, and the UV detector was set at 278 nm. About 5 µL of triacetate (1 mM) standard was also injected. The mass spectrometry detection was performed under negative electrospray ionization mode [ESI (−)]. The products of the enzymatic reactions, including dihydrophologlucinol, 3-hydroxy-5-oxo-hexanoate, triacetate, and acetoacetyl-CoA, were further verified by high-resolution LC-UV/MS (MaXis-ESI-QTOF). Acetone, the decarboxylation product of acetoacetate, was derivatized with 2,4-dinitrophenylhydrazine (55), followed by high-resolution LC-MS analysis. The LC separation was performed on Agilent ZORBAX Eclipse XDB-C18 (150 × 2.1 mm, product number 993700–902) column with a flow rate of 0.3 mL/min at RT. 0.1% Formate in water (solvent A) and acetonitrile (solvent B) was employed as mobile phase. A gradient of 0–5 min 0%−5% B, 5–10 min 5%−95% B, and 10–20 min 95% B was used. The sample injection volume was 5 µL, and the UV detector was monitored at 281 nm. 1 mM standards (dihydropholoroglucinol, triacetate, Acetoacetyl-CoA, and acetone-DNPH) were also analyzed. The mass spectrometry detection was performed under negative electrospray ionization mode [ESI (−)].

### Growth of *C. scatologenes* with phloroglucinol

*C. scatologenes* (DSM 757, ATCC 25775) was purchased from DSMZ (German Collection of Microorganisms and Cell Cultures). The rich medium was prepared by dissolving 10 g beef extract, 15 g casitone, 2.5 mL 1 N NaOH, 0.5 g yeast extract, 0.5 g K_2_HPO_4_, 50 µL 0.1% wt/vol Na-resazurin solution, 0.4 g D-glucose, 0.1 g cellobiose, 0.1 g maltose, 0.1 g starch (soluble), and 0.5 g/L L-cysteine hydrochloride in 100 mL distilled water and adjusting the pH to 7.0. To prepare the defined medium (56), 2.0 g KH_2_PO_4_, 2.0 g K_2_HPO_4_, 0.2 g MgC1_2_·6H_2_O, 5.0 g (NH_4_)_2_SO_4_, 25 mL 10% NaHCO_3_, 0.5 mL 0.1% wt/vol Na-resazurin solution, 128  mg nitrilotriacetic acid, 1 mg FeSO_4_·7H_2_O, 1 mg MnCl_2_·4H_2_O, 1.7 mg CoCl_2_·2H_2_O, 1 mg CaCl_2_·2H_2_O, 1 mg ZnCl_2_, 0.1 mg CuCl_2_, 0.1 mg H_3_BO_3_, 0.1 mg Na_2_MoO_4_·2H_2_O, 10 mg NaCl, 0.17 mg Na_2_SeO_3_, 0.26 mg NiSO_4_·6H_2_O, 1 mg Na_2_WO_4_·2H_2_O, 20 µg biotin, 20 µg folic acid, 100 µg pyridoxine-HCl, 50 µg thiamine-HCl, 50 µg riboflavin-HCl, 50 µg nicotinic acid, 50 µg pantothenic acid, 10 µg cyanocobalamin, 50 µg *p*-amino benzoic acid, and 50 µg lipoic acid were dissolved in 1 L distilled water. And add the following L-amino acids to a final concentration of 1 mM each: glycine, valine, leucine, isoleucine, methionine, histidine, arginine, phenylalanine, tyrosine, and tryptophan. After all supplements were added, 5% wt/vol cysteine HCI (pH 6.0) was added until reduction was effected (as judged by the discoloration of the resazurin). Cells were inoculated into the rich growth medium and cultivated anaerobically at 37°C for 2 d, spun down at 6,000 × *g* and resuspended in the defined medium. About 100 µL portions of the starter culture were transferred into three anaerobic bottles each containing 5.0 mL defined medium with none, 25 mM glucose, or 25 mM phloroglucinol, respectively. After 3–8 d of incubation at 37°C, the cultures with added glucose or phloroglucinol became turbid, indicating bacterial growth. The OD_600_ of the negative control was less than 0.08.

### LC-MS detection of products of phloroglucinol fermentation

The formation of acetate and butyrate during phloroglucinol fermentation was detected by LC-MS analysis using an Agilent 6420 Triple Quadrupole instrument. The 10 µL samples were chromatographed on an Agilent ZORBAX SB-C18 column (4.6 × 250 mm^2^, product number 880975–902) with a flow rate of 0.5 mL/min, eluting with 0.1% formic acid in water (solvent A) and 0.1% formic acid in acetonitrile (solvent B). A gradient of 0–4 min 0%−60% B, 4–8 min 60%–100% B, and 8–12 min 100% B was used. The mass spectrometry detection was performed under positive electrospray ionization mode [ESI (+)], and the analytes were monitored in multiple-reaction monitoring mode (57) .

### Protein identification by SDS-PAGE and mass spectrometry

Cells were harvested by centrifugation, lysed by boiling in Laemmli loading buffer, and analyzed on a 12.5% SDS-PAGE gel. Prominent protein bands induced by growth on phloroglucinol were manually excised. After in-gel digestion and extraction, the peptide mixtures were analyzed twice on an Orbitrap Fusion with EASY-nLC 1200 (Thermo Fisher Scientific). Resulting tandem mass spectra were searched against an appropriate protein database (retrieved from IMG) using Mascot (Matrix Science) and Proteom Discoverer V1.3 (Thermo Fisher Scientific) with “Trypsin” enzyme cleavage, static cysteine alkylation by chloroacetamide, and variable methionine oxidation. The peptide mass fingerprint search against the *C. scatologene*s (DSM 757) protein database GCF_000968375.1 by Proteome Discoverer (version 1.3) algorithm was used to identify proteins based on probability-based Mowse scoring algorithm with a confidence level of 95%.

### Real-time fluorescence quantitative PCR analyses

RNA was extracted from cultures that utilized either glucose or phloroglucinol + glucose as carbon sources, using RNAprep pure Cell/Bacteria Kit (TIANGEN). To perform reverse transcription, 600 ng RNA was added to a 20-µL reaction mixture containing 1× Hiscript III qRT SuperMix (Vazyme), according to the manufacturer’s recommended protocol. The reverse transcriptase was then inactivated at 85°C for 5 s, and the resulting cDNA was stored at −70°C. For a typical real-time fluorescence qPCR reaction, a 20-µL reaction mixture was prepared, containing 2 µL of 5× diluted cDNA, 0.2 µM gene-specific forward, and reverse primers and 1× AceQ Universal SYBR qPCR Master Mix (Vazyme). qPCRs were performed on an Applied Biosystems 7500. The primers were designed using Primer-BLAST (Table S2). All reagents and consumables for the experiments were RNase-free.

## Data Availability

Source data underlying Fig. 2–7, Fig. S3, Fig. S6, and Fig. S8-S12 are provided as a Source Data file. Other data are available from the corresponding authors upon reasonable request.

## SUPPLEMENTAL MATERIAL

Supplemental Information mbio.01099-23-s0001.pdf Tables S1 and S2; Fig. S1 to S12.

Data set S1 mbio.01099-23-s0002.xlsx Protein mass spectrometry results for band 1 to 4.

Data set S2 mbio.01099-23-s0003.csv cblaster search data.

Data set S3 mbio.01099-23-s0004.csv UniProt entries of the DPGCs.

Data set S4 mbio.01099-23-s0005.xlsx Presence of the phloroglucinol degradation pathway in phylogenetically and metabolically diverse bacteria.

ASM does not own the copyrights to Supplemental Material that may be linked to, or accessed through, an article. The authors have granted ASM a non-exclusive, world-wide license to publish the Supplemental Material files. Please contact the corresponding author directly for reuse.

## Abbreviations

SDR: short-chain dehydrogenase and reductase
PGR: phloroglucinol reductatse
DPGC: dihydrophloroglucinol cyclohydrolase
TfD: triacetate forming dehydrogenase ((S)-3-hydroxy-5-oxohexanoate dehydrogenase)
TAL: triacetate:acetoacetate-lyase
PG: phloroglucinol
DPG: dihydrophloroglucinol
