# Supplementary material for: Anaerobic phloroglucinol degradation by Clostridium scatologenes
Source: mBio. 2023 Jun 21;14(4):e01099-23. doi: 10.1128/mbio.01099-23 (PMC10470551; doi:10.1128/mbio.01099-23)
Supplement: Supplemental Information — Tables S1 and S2; Fig. S1 to S12. [file mbio.01099-23-s0001.pdf]

Supplementary Information

Anaerobic phloroglucinol degradation by *Clostridium scatologenes*

Yan Zhou[1,2,3*], Yifeng Wei[4], Li Jiang [5], Xinan Jiao[1,2,3*], Yan Zhang[5,6,7,8*]

From the [1]Jiangsu Key Laboratory of Zoonosis, Yangzhou University, Yangzhou, 225009, China.

[2]Key Laboratory of Prevention and Control of Biological Hazard Factors (Animal Origin) for Agrifood Safety and Quality, Ministry of Agriculture of China, Yangzhou University, Yangzhou, 225009, China.

[3]Jiangsu Co-Innovation Center for Prevention and Control of Important Animal Infectious Diseases and Zoonoses, Yangzhou University, Yangzhou 225009, Jiangsu Province, China.

[4]Singapore Institute of Food and Biotechnology Innovation, Agency for Science, Technology and Research (A*STAR), Singapore 138669, Singapore.

[5]Tianjin Key Laboratory for Modern Drug Delivery & High-Efficiency, Collaborative Innovation Center of Chemical Science and Engineering, School of Pharmaceutical Science and Technology, Tianjin University, Tianjin 300072, China.

[6]Frontiers Science Center for Synthetic Biology (Ministry of Education), Tianjin University, Tianjin 300072, China.

[7]Key Laboratory of Systems Bioengineering (Ministry of Education), Tianjin University, Tianjin 300072, China.

[8] Department of Chemistry, Tianjin University, Tianjin 300072, China.

[*]To whom correspondence should be addressed:

E-mail: yan.zhang@tju.edu.cn

E-mail: jiao@yzu.edu.cn

E-mail: yan_zhou@yzu.edu.cn

Table S1. Primers used to amplify *Cs*PGR, *Cs*DPGC and *Cs*TAL genes

Primers	From 5'-3'
*Cs*PGR-F	AACCTGTACTTCCAATCCAATATGGTTGATAAAAAACTTAT
CsPGR-R	GATCCGTTATCCACTTCCAATTTAGTTGGCCCAATATCCAC
*Cs*DPGC-F	AACCTGTACTTCCAATCCAATATGGGAAAGAAAGTATATGT
*Cs*DPGC-R	GATCCGTTATCCACTTCCAATTTAGAATCCACCATATGTGT
*Cs*TAL-F	AACCTGTACTTCCAATCCAATATGTCAATAAAAAAAAACAAT
*Cs*TAL-R	GATCCGTTATCCACTTCCAATTTATTTAGAAAAATTGCATA

Table S2. Primers used for qPCR experiments

Primer name	Sequence 5'-3'
PGR-F	TTCCAATTGGTCGCCTTGGT
PGR-R	TGGCCCAATATCCACCTTCTG
DPGC-F	ATGCGCCACGTCATGTTATG
DPGC-R	AACTCCCAACGTCCAGCTTT
TfD-F	TGCAGATATGGTTGGAACAGA
TfD-R	CCTTTACCGCTCTTGAACCCT
TAL-F	ATGGGGTGCCCTTGGAATTG
TAL-R	ACCCCAGCACTCCACATTAC
16S-F	CAGCTCGTGTCGTGAGATGT
16S-R	ACGTCATCCCCACCTTCCTC

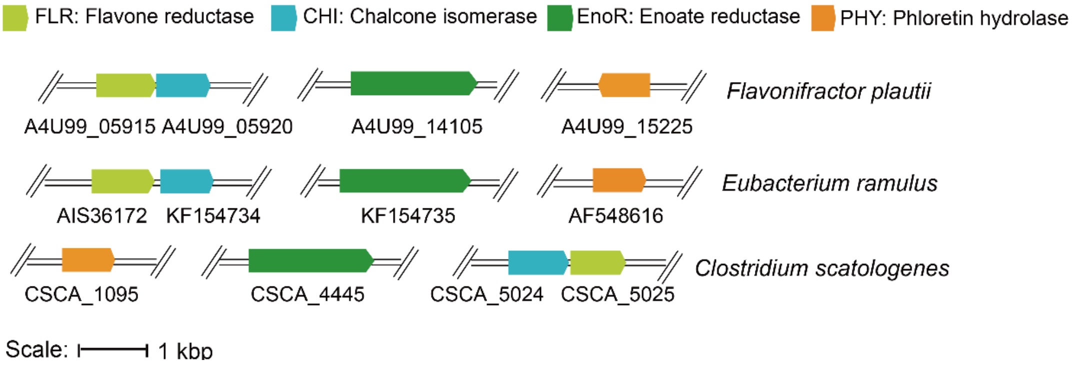

Fig. S1 Homologous genes involved in the degradation of flavonoids in *F. plautii*, *E. ramulus* and *C. scatologenes* respectively.

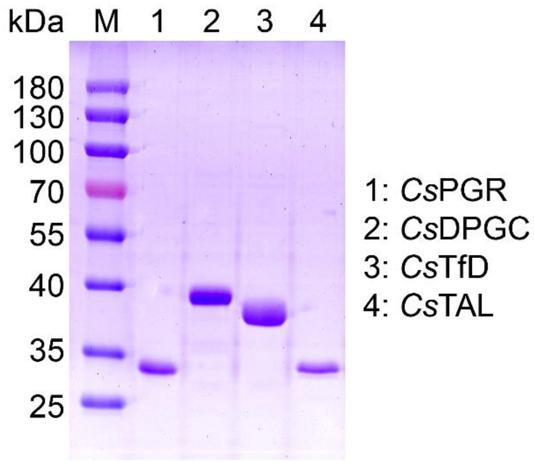

Fig. S2. SDS-PAGE gel analyses of recombinant proteins purified in this study.

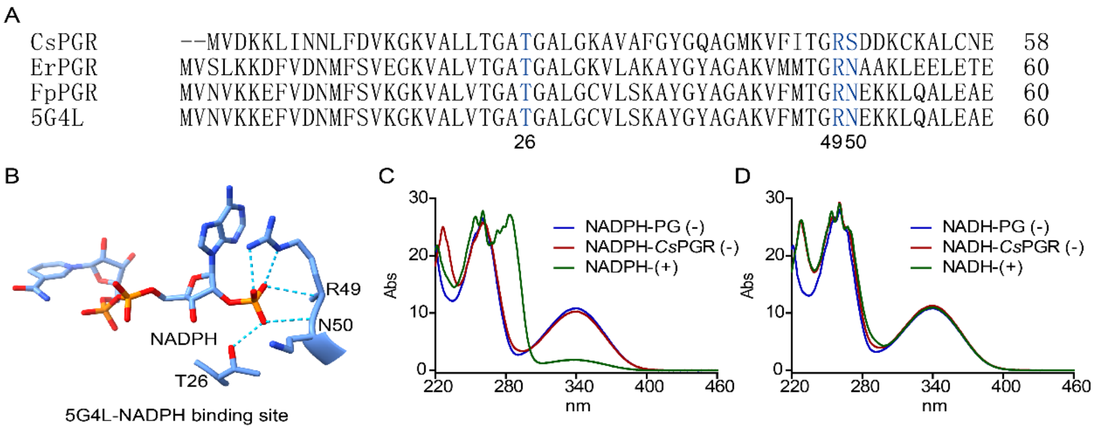

Fig. S3. ***Cs*PGR is a NADPH-dependent phloroglucinol reductase.** A) Sequence alignments of PGRs and a phloroglucinol reductase from *Clostridium* sp. ATCC BAA-442 (PDB ID:5G4L) [1]. The residues involved in interacting with the 2'-phosphate group of NADPH are colored in blue. B) The NADPH biding site in 5G4L, key residues involved in 2'-phosphate binding are displayed and labeled. C) UV-Vis spectra of *Cs*PGR activity assays with NADPH as the reductant. D) UV-Vis spectra of *Cs*PGR activity assays with NADH as the reductant. Negative controls omitted either phloroglucinol or *Cs*PGR in C) and D).

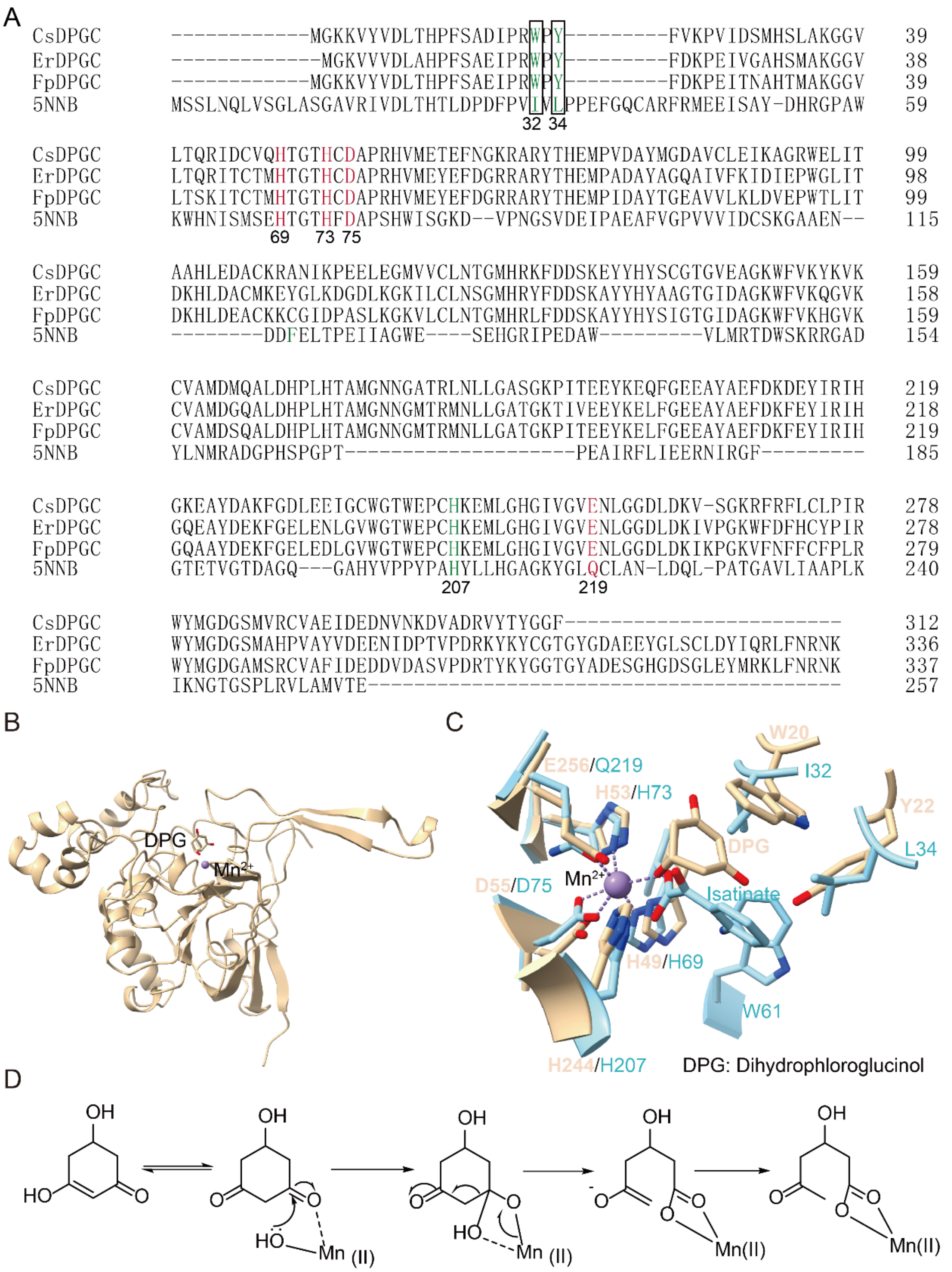

Fig. S4. Structural and functional analyses of *Cs*DPGC. A) Sequence alignments of DPGCs with isatin hydrolase from *Labrenzia aggregate* (PDB ID:5NNB) [2]. The conserved metal ligands are colored in red, and the residues involved in substrate interaction are colored in green. B) Homology model of *Cs*DPGC in complex with dihydrophloroglucinol (DPG) and Mn^{2+}. C) Superposition of the *Cs*DPGC active site (tan) with 5NNB active site (blue). Key residues involved in substrate binding are displayed and labeled. D) The proposed catalytic mechanism of DPGC.

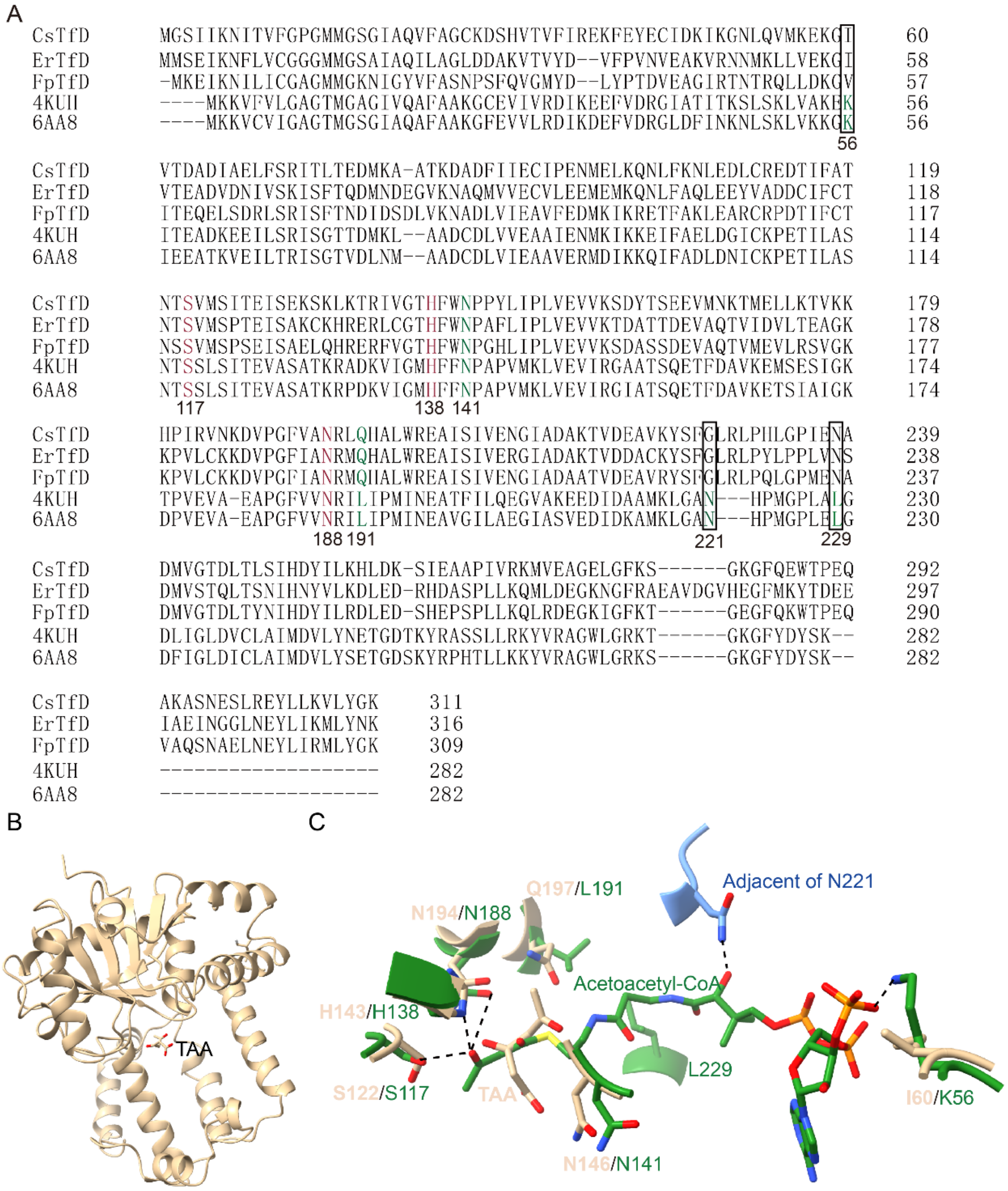

Fig. S5 Sequence and structure analyses of *Cs*TfD. A) Sequence alignments of TfDs with the (*S*)-3-hydroxybutylryl-CoA dehydrogenases from *Clostridium butyricum* (PDB ID:4KUH) [3] and *Clostridium acetobutylicum* ATCC 824 (PDB ID:6AA8) [4]. The residues involved in interaction with 2'-phosphate and pantothenic moiety of acetoacetyl-CoA are colored in green, and the residues involved in catalysis and stereospecific recognition are colored in red. B) Homology model of *Cs*TfD in complex with TAA (triacetate). C) Superposition of the *Cs*TfD active site (tan) with 4KUH active site (green, Asn221 of the opposing subunit of the dimer was colored in blue). Key residues involved in substrate binding are displayed and labeled, and the hydrogen bonds involved in substrate interaction are indicated by dashed line.

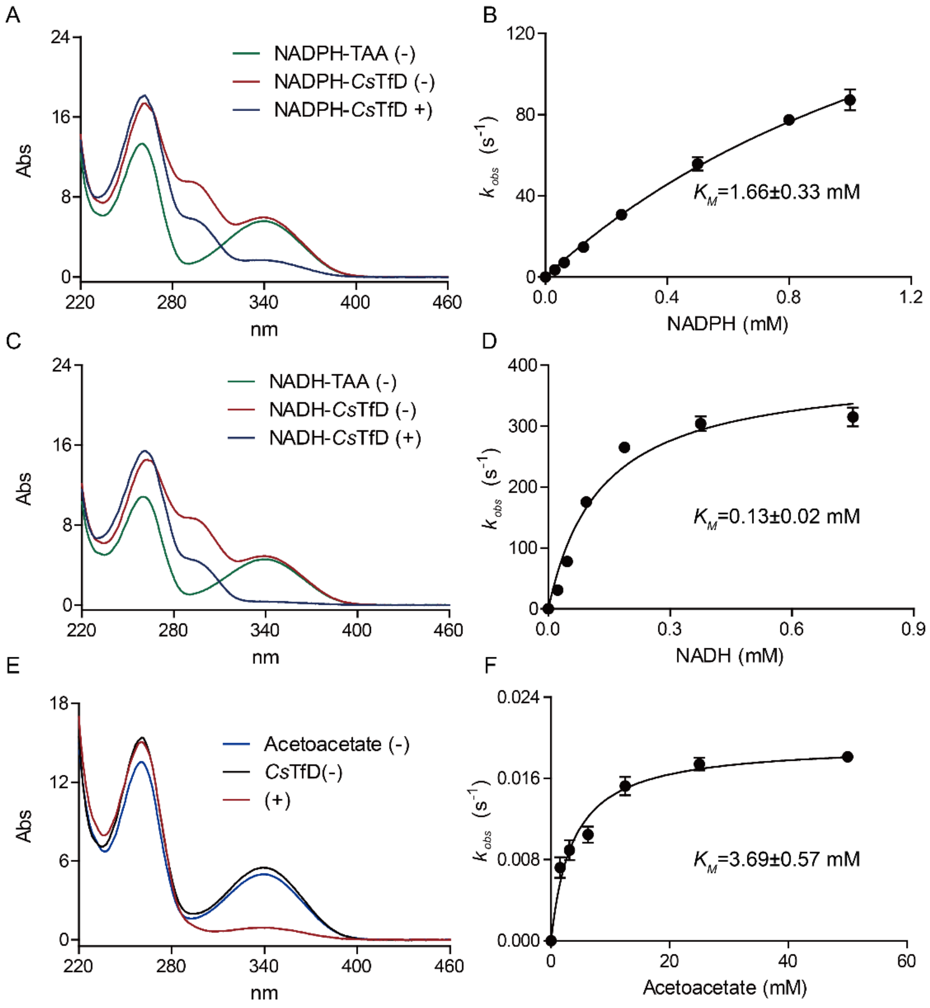

Fig. S6 *Cs*TfD enzyme activity assays. A) UV-Vis spectra of *Cs*TfD assays with NADPH as the reductant. Negative controls omitted TAA (triacetate) or *Cs*TfD. B) Michaelis-Menten kinetics of *Cs*TfD varying the concentrations of NADPH. C) UV-Vis spectra of *Cs*TfD assays with NADH as the reductant. Negative controls omitted triacetate or *Cs*TfD. D) Michaelis-Menten kinetics of *Cs*TfD varying the concentrations of NADH. E) UV-Vis spectra of *Cs*TfD assays with NADPH as the reductant and acetoacetate as the substrate. Negative controls omitted acetoacetate or *Cs*TfD. F) Michaelis-Menten kinetics of *Cs*TfD varying the concentrations of acetoacetate.

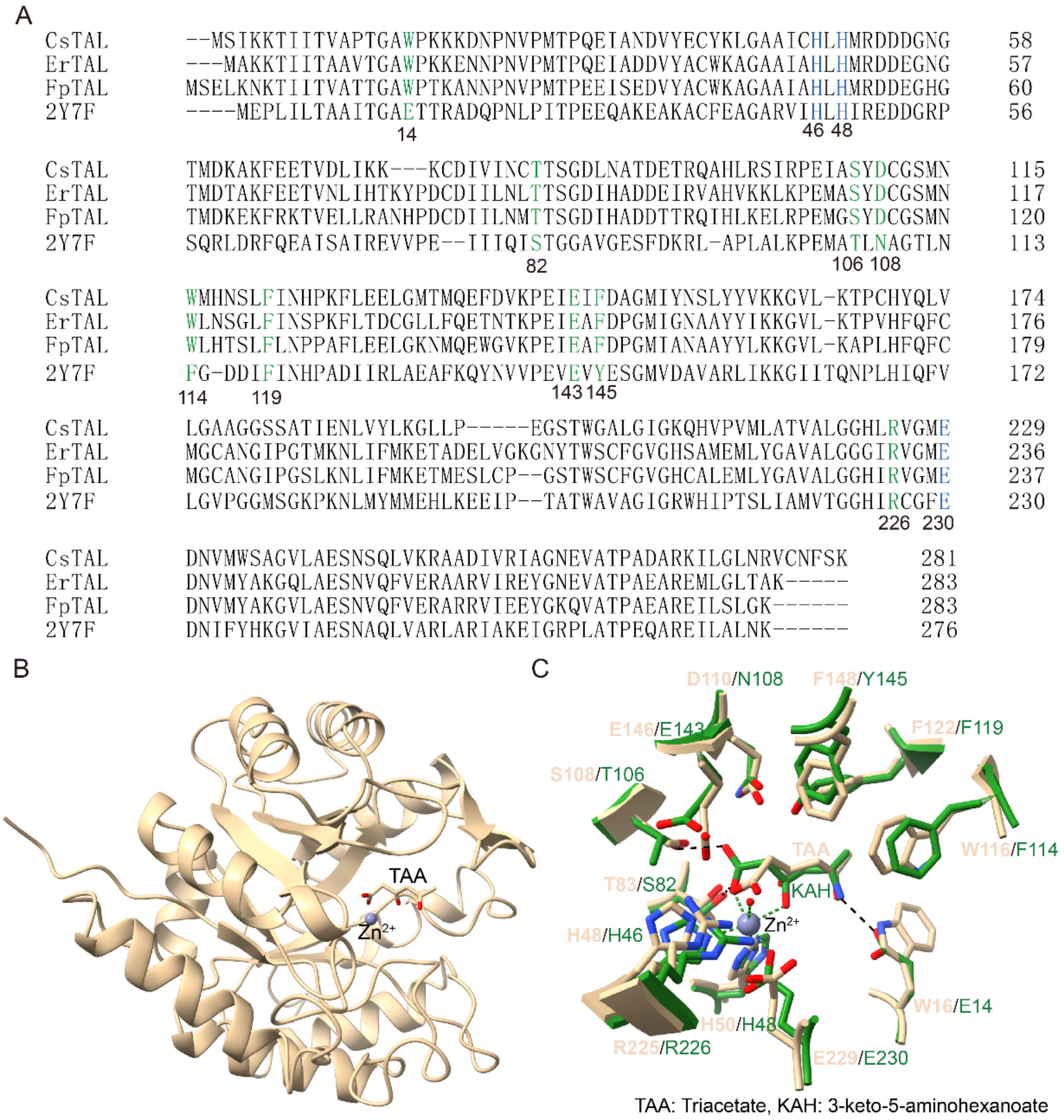

Fig. S7. Sequence and structure analyses of *Cs*TAL. A) Sequence alignments of TALs with the 3-keto-5-aminohexanoate cleavage enzyme from *Cloacimonas acidaminovorans* (PDB ID:2Y7F). The residues involved in substrate and Zn^{2+} interaction are colored in green and blue respectively. B) Homology model of *Cs*TAL in complex with TAA (triacetate) and Zn^{2+}. C) Superposition of the *Cs*TAL active site (tan) with 2Y7F active site (green). Key residues involved in substrate binding are displayed and labeled, and the hydrogen bonds involved in substrate interaction are indicated by dashed line.

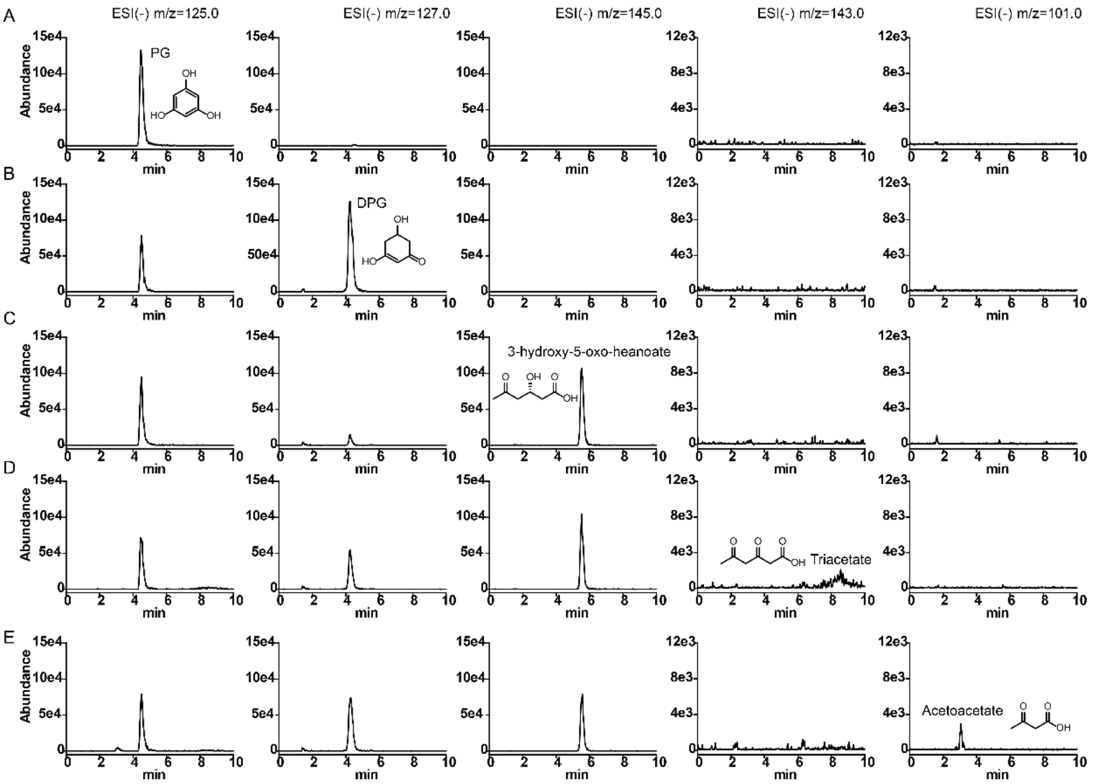

Fig. S8 *In vitro* reconstitution of the phloroglucinol degradation pathway. LC-MS analysis of reaction mixtures containing A) PG (phloroglucinol) and NADPH; B) PG, NADPH and *Cs*PGR; C) PG, NADPH, *Cs*PGR and *Cs*DPGC; D) PG, NADPH, *Cs*PGR, *Cs*DPGC and *Cs*TfD. E) PG, NADPH, *Cs*PGR, *Cs*DPGC, *Cs*TfD and *Cs*TAL.

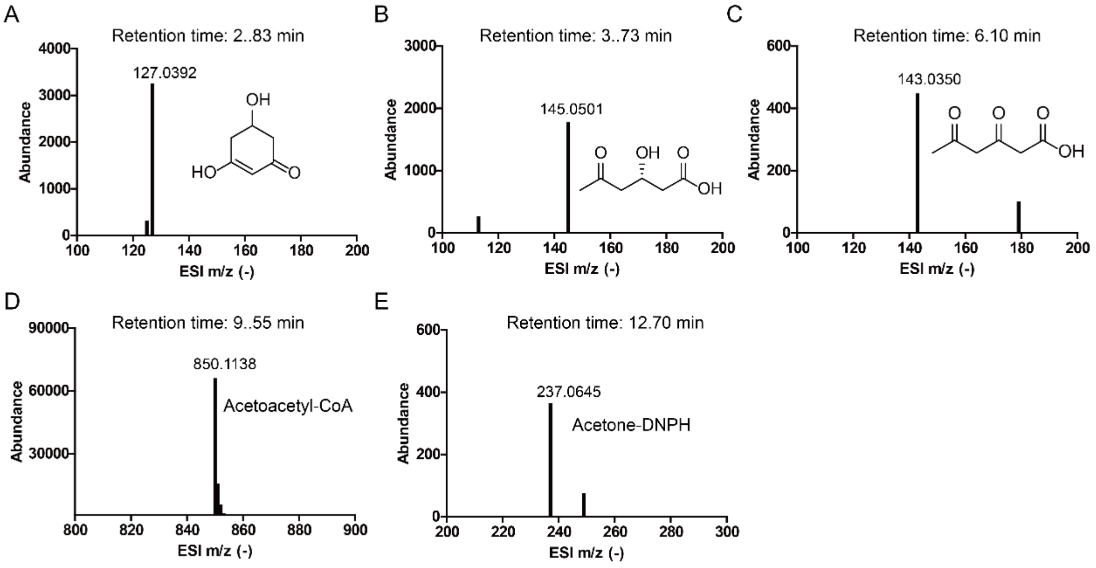

Fig. S9 The high resolution MS result of major products of enzymatic reactions. A) Negative ionization mass spectrum of the dihydrophloroglucinol peak in the assay eluted at 2.83 min. B) Negative ionization mass spectrum of the 3-hydroxy-5-oxo-hexanoate peak in the assay eluted at 3.73 min; C) Negative ionization mass spectrum of the triacetate peak in the assay eluted at 6.10 min; D) Negative ionization mass spectrum of the acetoacetyl-CoA peak in the assay eluted at 9.55 min. E) Negative ionization mass spectrum of the acetone-DNPH peak in the assay eluted at 12.70 min.

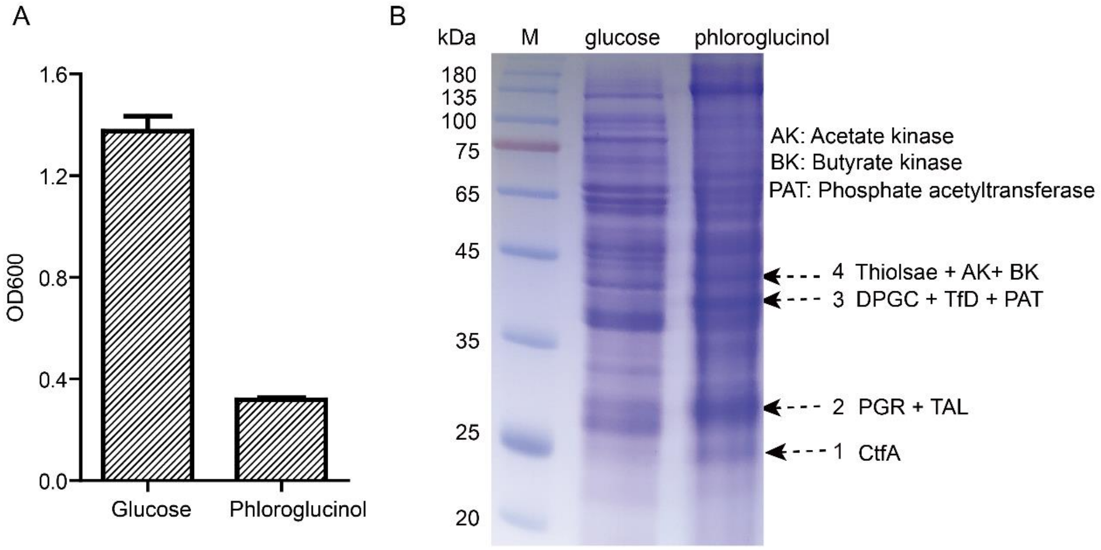

Fig. S10 Phloroglucinol supports the growth of *C. scatologenes* and induced SDS-PAGE A) Comparison of the growth of *C. scatologenes* on glucose and phloroglucinol. C) SDS-PAGE analysis of *C. scatologenes* grown on glucose and phloroglucinol. Source data are provided as **Supplementary data 1**.

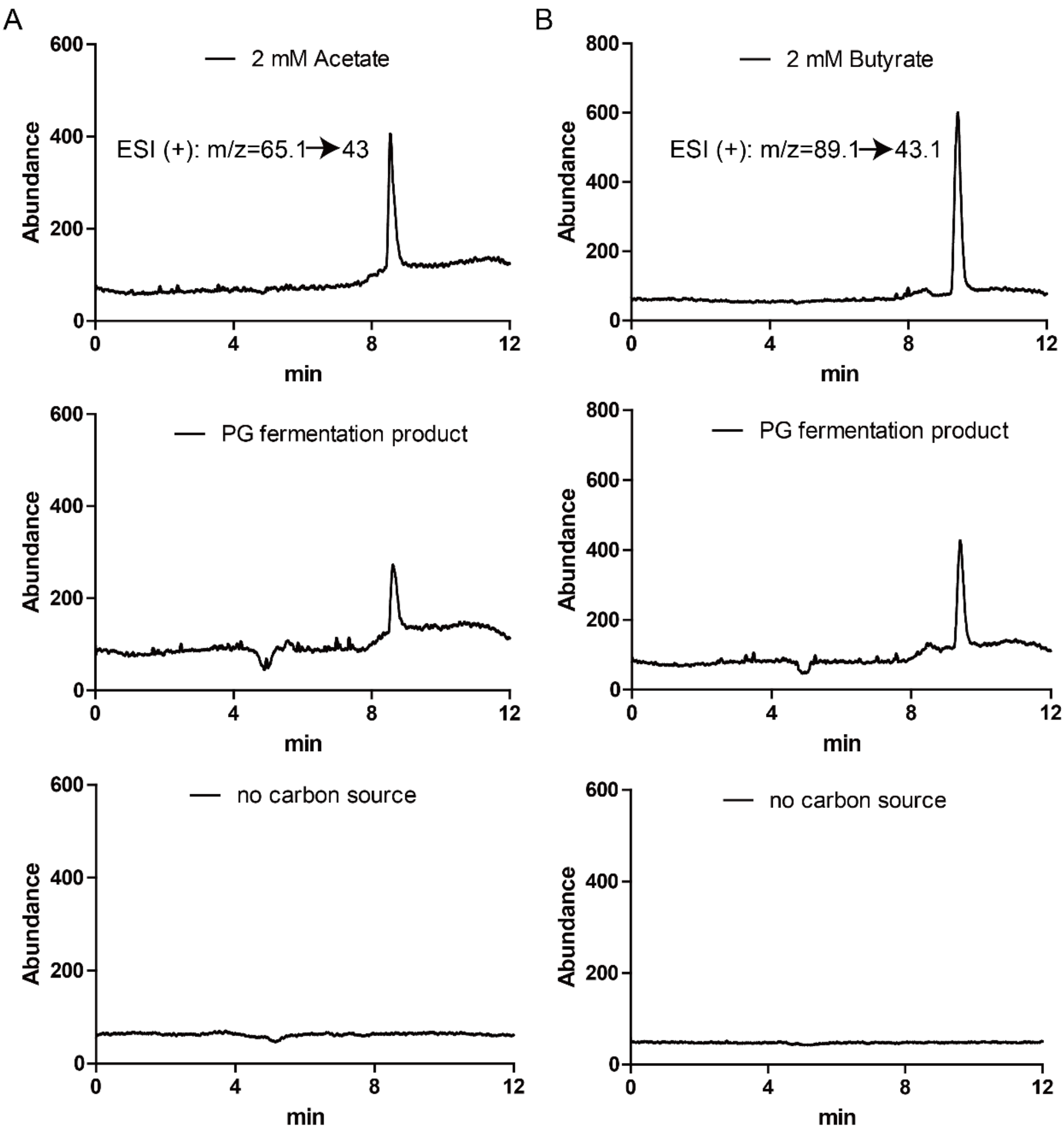

Fig. S11 Detection of acetate (A) and butyrate (B) in phloroglucinol fermentation products by LC-MS.

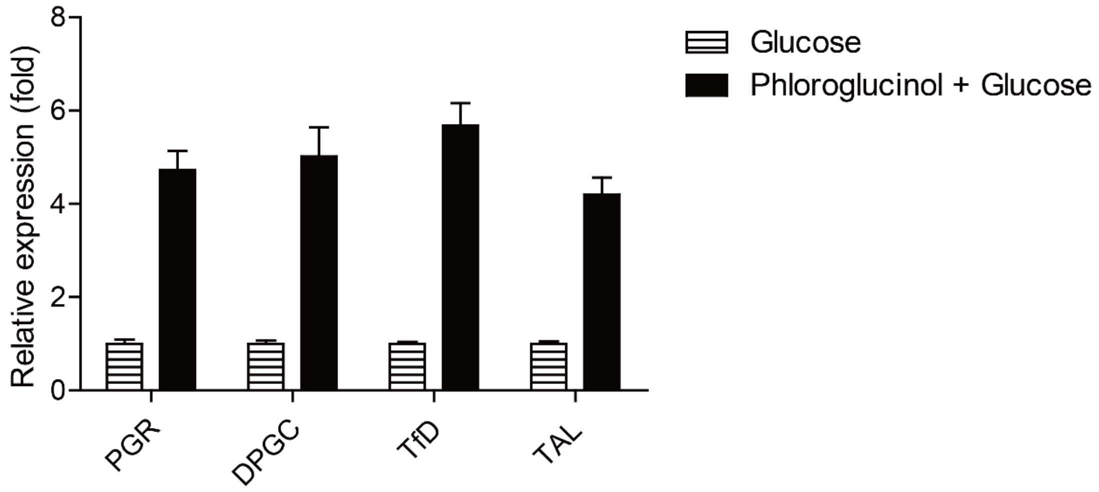

Fig. S12 qPCR analyses of the transcription levels of PGR, DPGC, TfD and TAL. The transcriptional levels of genes of interest were normalized by that of the 16S rRNA. The induction by phloroglucinol were displayed in comparison with the transcriptional data from glucose-grown cells. Bars in different samples were as indicated.

References

1. Conradt, D., Hermann, B., Gerhardt, S., Einsle, O. & Muller, M. Biocatalytic Properties and Structural Analysis of Phloroglucinol Reductases. *Angew Chem Int Ed Engl* **55**, 15531-15534 (2016).
2. Sommer, T. *et al.* A fundamental catalytic difference between zinc and manganese dependent enzymes revealed in a bacterial isatin hydrolase. *Sci Rep* **8**, 13104 (2018).
3. Kim, E.J. *et al.* Crystal structure of (S)-3-hydroxybutyryl-CoA dehydrogenase from Clostridium butyricum and its mutations that enhance reaction kinetics. *J Microbiol Biotechnol* **24**, 1636-1643 (2014).
4. Takenoya, M., Taguchi, S. & Yajima, S. Crystal structure and kinetic analyses of a hexameric form of (S)-3-hydroxybutyryl-CoA dehydrogenase from Clostridium acetobutylicum. *Acta Crystallogr F Struct Biol Commun* **74**, 733-740 (2018).
